# Special Notices

**Published:** 1886-12-04

**Authors:** 


### SPECIAL NOTICES.

The Word Puzzles and "Children's Corner" will be found on p. vi of the current number.

---

Hospital Secretaries will oblige by sending to the Editor a copy, in duplicate, of their Christmas appeals, and a statement of the deficiency they have to meet.

---

### JOURNALISTIC ENTERPRISE EXTRAORDINARY.

The article by the Prime Minister, which we published last week, naturally excited a great deal of interest. The Press Association, with its usual enterprise and despatch, called upon us on Wednesday, 24th ult., and arrangements were then made to telegraph the article to the provincial press, as well as to supply copies to the London daily and evening papers on Thursday evening, so that all might be placed upon equal terms as to republication. It is, of course, usual, for a weekly or monthly publication to make similar arrangements in regard to contributions of special interest and importance, providing at the same time that such republication shall not anticipate in point of time the appearance of the original article. English editors have always shown the fairest regard to the rights and property of their neighbours, and any infringement of such rights is almost, if not quite, unknown in this country. In these circumstances, much comment has been caused by the publication of an unauthorised and incomplete copy of Lord Salisbury's article in the fifth edition of *The Globe* of Wednesday, 24th ult., before it had been passed for the press, and of course long before the publication of the edition of The Hospital in which the original article was to appear. The unauthorised publication of Lord Salisbury's article by *The Globe*, under such circumstances, has done serious injury to this Journal; but as the matter may come before the Courts, we do not feel in a position to state in detail, at present, how *The Globe* obtained possession of that which they published. We can only express our feelings of astonishment, that any English newspaper, which had inadvertently done, what *The Globe* did in this case, should not spontaneously make ample apology and express regret, on learning the facts, unless the transaction from first to last had the approval and sanction of its editor and proprietors.

